# *RNY3* modulates cell proliferation and *IL13* mRNA levels in a T lymphocyte model: a possible new epigenetic mechanism of IL-13 regulation

**DOI:** 10.1007/s13105-022-00920-6

**Published:** 2022-09-12

**Authors:** Miguel Estravís, Asunción García-Sánchez, Maria J. Martin, Jacqueline Pérez-Pazos, María Isidoro-García, Ignacio Dávila, Catalina Sanz

**Affiliations:** 1 Instituto de Investigación Biomédica de Salamanca (IBSAL), Salamanca, Spain; 2 Red Cooperativa de Investigación en Salud–RETICS ARADyAL, ISCIII, Madrid, Spain; 3 Departamento de Ciencias Biomédicas y del Diagnóstico, Universidad de Salamanca, Salamanca, Spain; 4 Departamento de Bioquímica y Biología Molecular, Universidad de Salamanca, Salamanca, Spain; 5 Unidad de Farmacogenética y Medicina de Precisión, Servicio de Bioquímica Clínica, Servicio de Alergología, Hospital Universitario de Salamanca, IBSAL, Salamanca, Spain; 6 Servicio de Bioquímica Clínica, Complejo Asistencial Universitario de Salamanca, Salamanca, Spain; 7 Departamento de Medicina, Universidad de Salamanca, Salamanca, Spain; 8 Servicio de Inmunoalergia, Complejo Asistencial Universitario de Salamanca, Salamanca, Spain; 9 Departamento de Microbiología y Genética, Universidad de Salamanca, Salamanca, Spain

**Keywords:** Y-RNA, Interleukin 13 (IL-13), Gene expression, Epigenetics, Lymphocyte activation, Jurkat cells

## Abstract

Allergic asthma is the most common type of asthma. It is characterized by T_H_2 cell–driven inflammation in which interleukin-13 (IL-13) plays a pivotal role. Cytoplasmic RNAs (Y-RNAs), a variety of non-coding RNAs that are dysregulated in many cancer types, are also differentially expressed in patients with allergic asthma. Their function in the development of the disease is still unknown. We investigated the potential role of *RNY3* RNA (hY3) in the T_H_2 cell inflammatory response using the Jurkat cell line as a model. hY3 expression levels were modulated to mimic the upregulation effect in allergic disease. We evaluated the effect of hY3 over cell stimulation and the expression of the T_H_2 cytokine *IL13*. Total RNA was isolated and retrotranscribed, and RNA levels were assessed by qPCR. In Jurkat cells, hY3 levels increased upon stimulation with phorbol 12-myristate 13-acetate (PMA) and ionomycin. When transfecting with high levels of hY3 mimic molecules, cell proliferation rate decreased while *IL13* mRNA levels increased upon stimulation compared to stimulated control cells. Our results show the effect of increased hY3 levels on cell proliferation and the levels of *IL13* mRNA in Jurkat cells. Also, we showed that hY3 could act over other cells via exosomes. This study opens up new ways to study the potential regulatory function of hY3 over IL-13 production and its implications for asthma development.

**Supplementary Information:**

The online version contains supplementary material available at 10.1007/s13105-022-00920-6.

## Introduction

Allergic diseases have become a global public health issue in the last decades, affecting both developed and developing countries. Allergies are caused by hypersensitivity of the immune system to substances termed allergens that are perceived as a threat and otherwise would be considered harmless. They produce the characteristic type 2 immune response, with a subsequent inflammatory process, that, when affecting lower airways, produces allergic asthma, the most common type of asthma.

Allergic asthma is a chronic inflammatory disorder of the lung airways causing airflow obstruction, which can be triggered by airborne allergens such as house dust mites, pollen from grass, weeds, trees, animal dander, or fungal spores. Classically, this form of asthma is associated with an increase in serum immunoglobulin E (IgE) antibodies and eosinophilia, as well as the presence of interleukin 4 (IL-4)–, IL-5-, and IL-13-producing cells in bronchoalveolar lavage (BAL) [18]. Among them, IL-13 has been shown to play a central role in asthma, mediating many of its key physiologic and pathological features [4]. Its actions in asthma include switching plasma cell antibody synthesis from IgM to IgE production, promoting migration of eosinophils into the lung, increasing permeability and sloughing of airway epithelial cells, increasing mucus production, production of inducible nitric oxide synthase by airway epithelial cells, the transformation of airway fibroblasts into myofibroblasts, proliferation of airway smooth muscle, and stimulation of airway hyperresponsiveness, among others [4]. Despite the signaling cascades that lead to *IL13* transcription being well known [35], little is known about the post-transcriptional regulation of *IL13*. Although initially it was suggested that HuR—an RNA stabilizing protein—regulated post-transcriptionally *IL13*, stabilizing *IL13* mRNA upon cell activation in Jurkat cells [3], and that HuR regulated mRNA stability through the 3′ UTR of different cytokine transcripts [29], Karginov et al. showed that HuR indeed exerted its control through upstream factors during T cell transcription activation [13]*.*

Like other allergic diseases, allergic asthma represents a clear example of a multifactorial disorder, in which predisposing genetic variants cannot explain its rapid prevalence increase. Immunologic development and different environmental cues, like changes in lifestyle, hygiene, or access to antibiotics, have been proposed to be essential factors in allergic asthma development [26]. In recent years, epigenetics has emerged as the underlying mechanism linking genetics, development, and environmental triggers for the onset and progression of allergic diseases and asthma [10]. Thus, several mechanisms have been described to fine-tune different processes, such as gene expression or protein translation. In fact, small non-coding RNAs (sncRNAs) are involved in the epigenetic regulation of several allergic diseases, and many micro-RNAs—a type of sncRNAs—have been associated with allergic asthma by a large number of studies [30].

Notwithstanding, investigating other non-coding RNAs (ncRNAs) in allergy is still at an early stage. In a previous transcriptomic study performed with allergic asthmatic patients, we found differential transcription patterns between allergic asthmatic patients and controls, with most of the top 50 differentially expressed transcripts being a kind of ncRNAs called Y-RNA [23]. Recently, we have validated the overexpression of these Y-RNAs in a broader population of 208 allergic patients compared with 96 non-allergic controls and performed an in silico analysis of their potential RNA targets where several genes associated with immune response pathways were identified [11]. This family of sncRNAs was named c**y**toplasmic RNAs (Y-RNAs) as opposed to the n**u**clear RNA (U-RNAs) family, both first described as components of the ribonucleoprotein complexes targeted by autoantibodies in patients with systemic erythematous lupus [19].

Y-RNAs have a size that varies between 75 and 115 nucleotides, and although they differ slightly in their primary and secondary structures, they have a typical structure consisting of a double helix stem with paired bases, formed by the 5ʹ and 3ʹ ends of the RNA. Near the 5ʹ end, an unpaired cytosine forms a bulge in the structure necessary for its recognition by Ro60 [28]. Four different Y-RNAs have been described in humans: RNY1, RNY3, RNY4, and RNY5. Also, over 1000 pseudogenes exist that are the product of relatively recent reverse transposition events, with around 90% identity with some of the four Y-RNAs [24].

Even though little is known about its biology, two functions of these RNAs have been described. The best known is to form part of a ribonucleoprotein complex with Ro60 and other proteins. Functional studies of this complex suggest a role in the quality control of non-coding RNAs that require unique folding, i.e., rRNAs or tRNA, in mRNA processing and also the binding to misfolded 3ʹ ends of free RNA. Another function of the Y-RNAs described to date is related to DNA replication and does not rely on the association with the Ro60 ribonucleoprotein complex. [6]. In addition to the functions described, their presence in extracellular fluids, from saliva to blood serum, either as part of ribonucleoprotein complexes or associated with extracellular vesicles, may indicate a role in signal transmission, amplification, or modulation of different responses in their cellular targets both at the local and systemic level [6]. While the number of studies on the effects of extracellular Y-RNA remains limited, they have been described as involved in regulating immune responses and associated with both pro- and anti-inflammatory effects [6, 9].

The role of Y-RNAs in immune system regulation is, therefore, beginning to be understood. Here we aimed to explore the function of these small non-coding RNAs in the modulation of the allergic response and the subsequent inflammatory processes using the Jurkat E6-1 cell line as a model for T cells, an essential cell type for the allergic response.

## Materials and methods

### Cell culture

Human Jurkat T cell line (E6-1 clone from American Type Culture Collection) was cultured in RPMI medium supplemented with l-glutamine, charcoal-stripped fetal bovine serum (FBS) at a final concentration of 10%, and penicillin/streptomycin at a final concentration of 1%. Cells were cultured at 37 °C in an air atmosphere containing 5% CO_2_. When required for colorimetric assays, RPMI medium without phenol red was used. Cells were maintained at a concentration between 1 × 10^5^ and 1 × 10^6^ viable cells per mL, and fresh medium was added every 2 to 3 days. Experiments were performed in triplicate.

### Cell stimulation

Jurkat cells were simultaneously stimulated with phorbol 12-myristate 13-acetate (PMA) and ionomycin for 24 h [1]. Briefly, cells were incubated in the presence of PMA 10 ng/mL and ionomycin 1 µM. As a control, DMSO was added to untreated cells at the same amount used in PMA and ionomycin. After stimulation, cells were harvested, and media were recovered and stored at − 20 °C for analysis.

### Cell proliferation assay

The Amersham Cell Proliferation BioTrak ELISA System kit was used (GE Healthcare, Chicago, IL, USA) to assess cell proliferation according to the manufacturer’s guidelines. It consists of a bromodeoxyuridine (BrdU) labeling. Media were removed from cells and incubated overnight in the presence of BrdU before fixation. Then, cells were blocked, incubated with peroxidase-labeled anti-BrdU, and washed before reacting with TMB (3,3ʹ,5,5ʹ-tetramethylbenzidine) substrate. The reaction was stopped with 1 M sulfuric acid when color development was sufficient for optical density measurement. Plates were read within 10 min after stopping the reaction in a Multiskan Ascent Plate Reader (Thermo Fisher Scientific, Waltham, MA, USA).

### Transient transfection

**Fig. 1:**
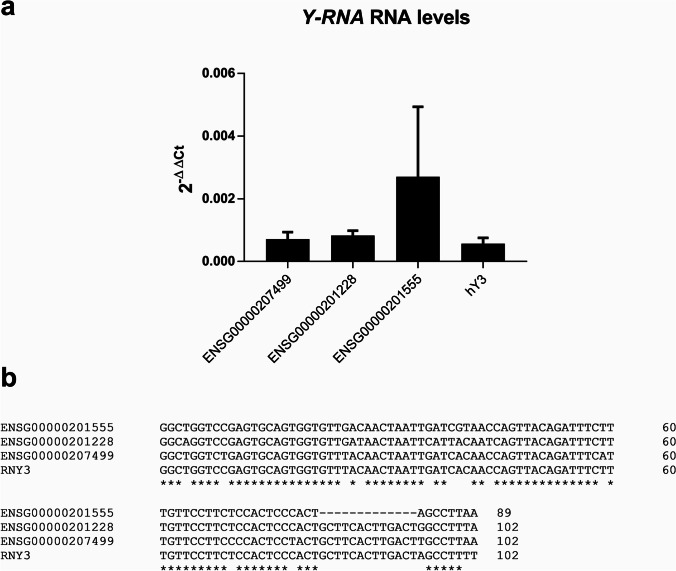
**a** Y-RNA expression assessed by RT-qPCR in Jurkat cells growing in RPMI media supplemented without any stimulus. **b** Sequence alignment of Y-RNAs overexpressed in allergic patients and *RNY3*

Jurkat cells were transfected with a synthetic *RNY3* RNA (hY3), consistent in 102-mer RNA with an identical sequence to human *RNY3* RNA (NCBI Reference Sequence: NR_004392.1; Fig. 1b) provided by Integrated DNA Technologies (Coralville, IA, USA). According to the manufacturer’s instructions, the transfection was performed with Xfect™ RNA Transfection Reagent (TaKaRa Bio, Shiga, Japan). Briefly, 5 × 10^5^ cells per mL were plated in RPMI medium without FBS, and 50 pmol of RNA per mL was used for optimal transfection. RNA reaction buffer, RNA, and Xfect™ RNA Transfection polymer complexes were incubated for 10 min before adding to cultures. Cells were incubated for 4 h at 37 °C in a CO_2_ incubator and then centrifuged to remove nanoparticle complex–containing media. Fresh medium with charcoal-stripped FBS was added to the cells, and then, they were incubated for 48 h before the gene and protein expression assays. Control cells were transfected in parallel with IDTE buffer alone (Integrated DNA Technologies, Coralville, IA, USA). Experiments were performed in triplicate. Average and standard deviation were used for analysis and interpretation.

### RNA isolation

Total RNA was isolated using the RNeasy Plus Mini kit (Qiagen, Hilden, Germany) and DNase treated (Qiagen, Hilden, Germany). Reverse transcription (RT) was performed on 1000 ng of total RNA using Superscript III First-Strand Synthesis System for RT-PCR (Invitrogen, Carlsbad, CA, USA).

### Gene expression analysis

Relative qPCR was performed as previously described [21, 34]. Briefly, the LightCycler480 Instrument was used with SYBR Green I Master (Roche, Basel, Switzerland). Fold induction was calculated by the comparative Ct method using the 2^−(ΔΔCt)^ or 2^− (ΔCt)^ formulas [21]. *GAPDH* was used as the reference gene. The experiments were replicated three times, and every sample was performed in triplicate, with non-template controls and a calibrator. All procedures were performed following MIQE guidelines [2]. Table 1 lists the primers used in this study.

**Table 1:** Primer sequence lists used for RT-qPCR

Name	Sequences (5′-3′)	Product (bp)
ENSG00000201555-F	CGAGTGCAGTGGTGTTGAC	76
ENSG00000201555-R	GCTAGTGGGAGTGGAGAAGG	
ENSG00000201228-F	AGCAGGTCCGAGTGCAGT	97
ENSG00000201228-R	GCCAGTCAAGTGAAGCAGTG	
ENSG00000207499-F	CTGGTCTGAGTGCAGTGGTG	100
ENSG00000207499-R	TTAAGGCAAGTCAAGTGAAGCA	
hY3-F	GTCCGAGTGCAGTGGTGTTTAC	93
hY3-R	GGCTAGTCAAGTGAAGCAGTGG	
GAPDH-F	CTCTGCTCCTCCTGTTCGAC	204
GAPDH-R	ACGACCAAATCCGTTGACTC	
IL2-F	CACAGCTACAACTGGAGCATTTA	96
IL2-R	ATGTGAGCATCCTGGTGAGTT	
IL13F	CTGGAATCCCTGATCAACGTG	115
IL13-R	GGACATGCAAGCTGGAAAACT	
GATA3-F	TCTGACCGAGCAGGTCGTA	143
GATA3-R	GCGACGACTCTGCAATTCTG	

### Cytokine quantification

Cell supernatants were harvested, centrifuged (400 g, 10 min), and stored at − 80 °C. According to the manufacturer’s guidelines, cytokine levels were determined using Human Cytokine Uncoated ELISA kits provided by Invitrogen (Carlsbad, CA, USA). After incubation, plates were read in a Multiskan Ascent Plate Reader (Thermo Fisher Scientific, Waltham, MA, USA).

### Exosome purification

According to the manufacturer’s indications, exosomes delivered to culture media were purified with ExoQuick TC® ULTRA EV Isolation Kit for Tissue Culture Media (System Biosciences, Palo Alto, USA), and a fraction was used to assess purification by Western blot using the exosome markers CD81, CD9, and CD63 (EXO AB kit, System Biosciences) (Supp. Fig. 1). Western blot was performed as described previously [32]. Cells were harvested after the indicated incubation times and centrifuged (400 g) for 5 min. Cell pellets were stored for later processing, and the cell culture media supernatant was collected and stored at − 80 °C until processing. Five milliliters of media were used to purify exosomes. Exosome purification was assessed by Western blot using exosome antibodies provided by System Biosciences (Palo Alto, USA). As a control, media containing charcoal-stripped FBS were purified in parallel.

### Statistical analysis

Experiments were performed in triplicate. The average and standard deviation (SD) of the different measurements were calculated. Levene’s test was used to assess sample homoscedasticity, and statistical differences were calculated by *t*-test for independent samples. Statistical significance for the tests was established at 0.05. Statistical analysis was performed using SPSS software (version 23) (IBM, Armonk, NY, USA).

## Results

### Jurkat cell line expresses *RNY3*

A previous transcriptomic analysis by our group found Y-RNAs as the most abundant group of differentially expressed non-coding RNAs in allergic patients [23], and three of them were analyzed in an independent population of allergic patients and healthy controls [11]. These results prompted us to investigate the role of Y-RNAs in the immunologic response observed in allergic patients. To explore the physiological effects of Y-RNA modulation, we decided to use the Jurkat E6-1 cell line as an in vitro model for T cells, as this cell type plays an essential role in the allergic response. Jurkat is an immortalized human T lymphocyte cell that has been widely utilized as a cell model to understand the immune responses associated with T cell signaling.

We could confirm by qPCR that the three Y-RNAs described in our previous publications were expressed at a similar magnitude range when normalized with the reference gene *GAPGH* (Fig. 1a). As these three Y-RNAs shared high homology with the *RNY3* consensus sequence (Fig. 1b), one of the four types of Y-RNAs described in humans, the possibility of finding interference effects among them led us to deepen the study of *RNY3* (hY3) general mechanisms in our model.

### hY3 levels increase in stimulated cells

**Fig. 2:**
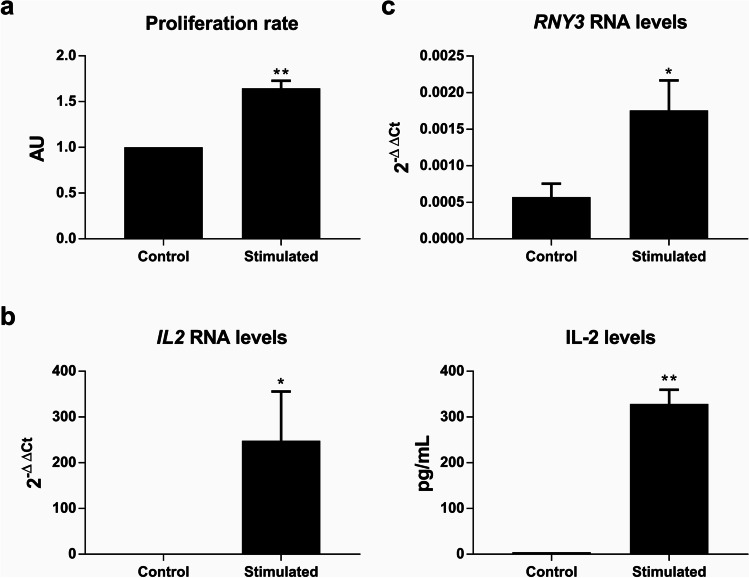
*RNY3* is overexpressed under stimulation of Jurkat cells. **a** Proliferation index in cells stimulated with PMA and ionomycin compared with untreated cells. **b** *IL2* mRNA levels were measured by RT-qPCR (left), and IL-2 protein secreted to the media by ELISA (right) in stimulated and unstimulated cells. **c** hY3 RNA levels were measured by RT-qPCR in unstimulated and stimulated cells. Proliferation was assessed by BrdU incorporation (AU, arbitrary units; *p < 0.05; **p < 0.01)

Our first approach to study the possible role of hY3 in Jurkat physiology was to analyze hY3 levels upon stimulation. We used PMA and the calcium ionophore ionomycin to stimulate Jurkat cells, a well-established model for studying T cell activation [1]. When cells were stimulated, cell proliferation increased significantly as per BrdU incorporation (*p* = 0.005) (Fig. 2a). Under these conditions, both *IL2* mRNA levels (*p* = 0.016) and IL-2 protein levels (*p* = 0.003) were strongly increased after 24 h (Fig. 2b), supporting the notion that the T cells were correctly stimulated under our experimental conditions [1]. The analysis of hY3 levels in those cells showed a significant increase (*p* = 0.01) upon stimulation (Fig. 2c).

### Effect of increased amounts of hY3 on cell proliferation

**Fig. 3:**
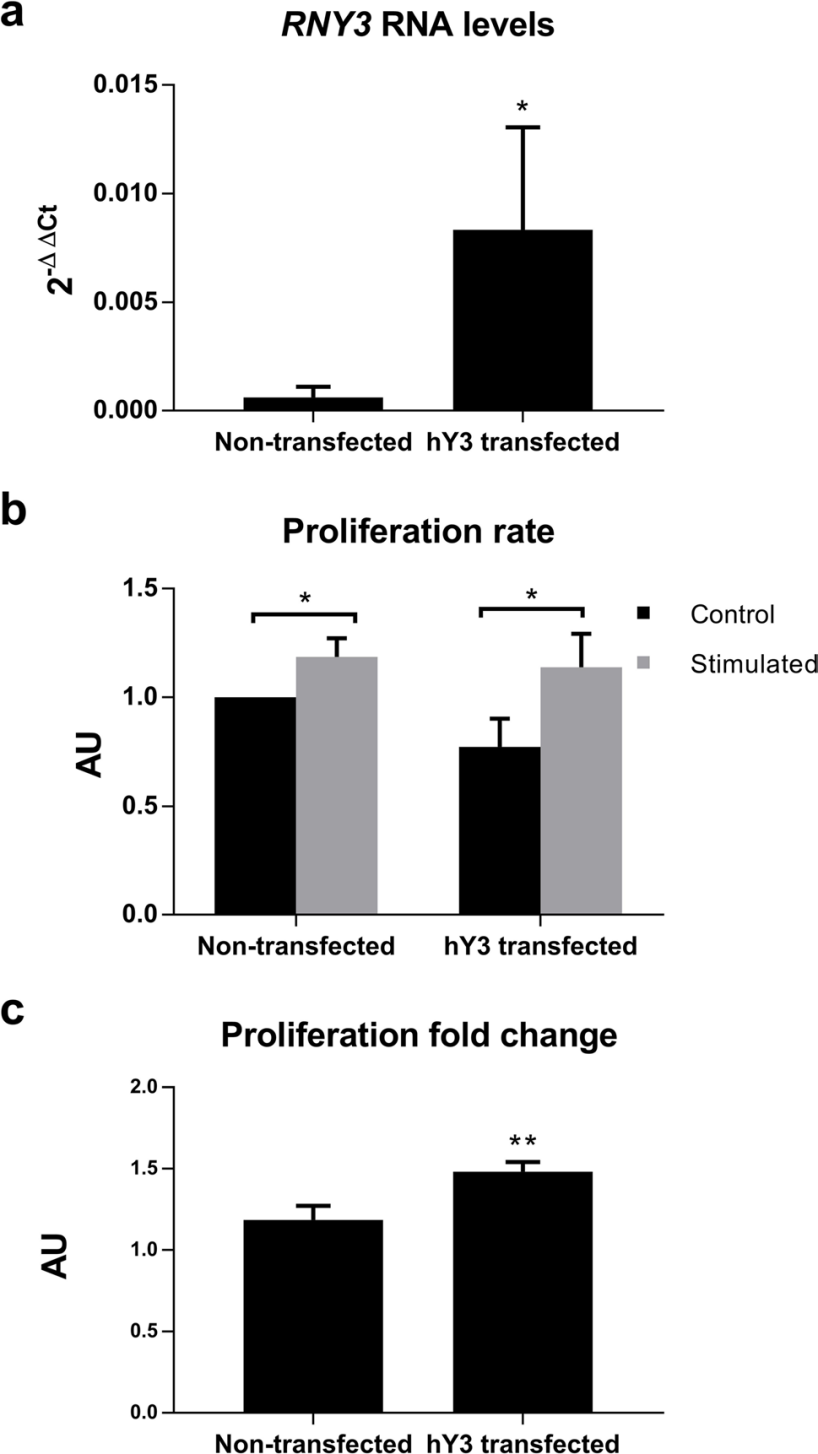
hY3 transfection affects cell proliferation. **a** hY3 RNA levels were measured by RT-qPCR after transfection. **b** Comparison of proliferation rates of cells transfected with hY3 and the control upon stimulation with PMA and ionomycin for 24 h and in normal conditions. **c** Proliferation fold change was calculated as the proliferation of cells stimulated with PMA and ionomycin for 24 h divided by the proliferation of unstimulated cells. Proliferation was assessed by BrdU incorporation. (AU, arbitrary units; *p < 0.05; **p < 0.01)

A previous publication from our group showed an increased expression of three Y-RNAs in peripheral blood from allergic asthmatic patients compared to healthy individuals [11]. To investigate the functional effects of increased Y-RNA levels on Jurkat cells, we transfected them with synthetic hY3. After 48 h, a significant increase in the intracellular gene expression levels of hY3 (*p* = 0.048) could be detected by qPCR (Fig. 3a). Evaluating cell proliferation rates (Fig. 3b), we found reduced but non-significant levels (*p* = 0.094) in cell proliferation of hY3-transfected cells when comparing control non-transfected vs. control hY3-transfected cells.

No significant differences in cell proliferation rates were found upon stimulation between non-transfected and hY3-transfected cells (Fig. 3b). However, the fold change (control vs. stimulated) was significantly higher (*p* = 0.008) in hY3-transfected cells (Fig. 3c).

### hY3 levels affect *IL13* mRNA levels upon stimulation

**Fig. 4:**
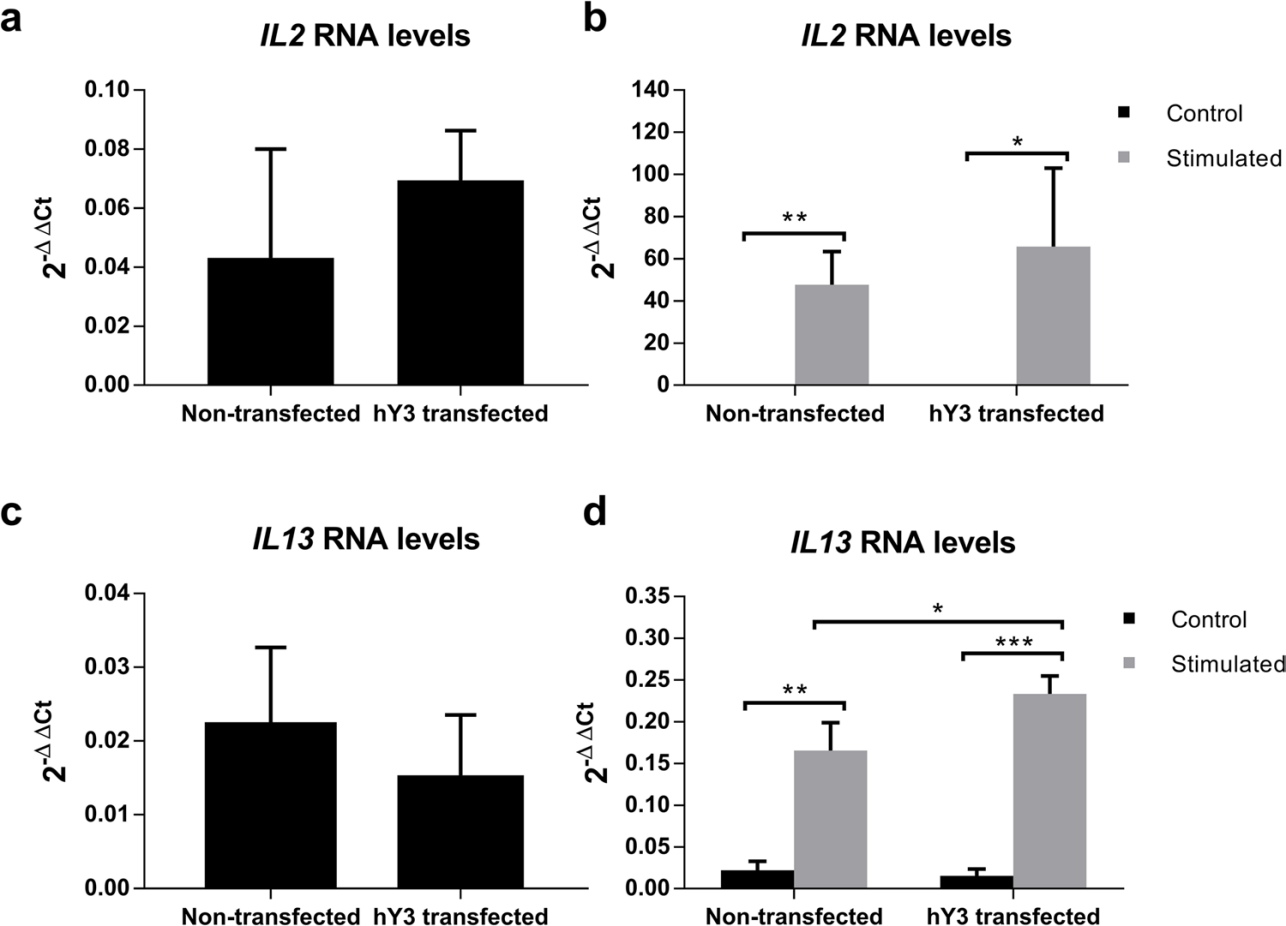
hY3 transfection effect over *IL13* and *IL2* mRNA levels. **a** *IL2* mRNA levels were measured in cells transfected with hY3 compared with cells not transfected (ns). **b** *IL2* mRNA levels were measured in cells stimulated with PMA and ionomycin for 24 h compared to cells not stimulated (ns). **c** *IL13* mRNA levels were measured in cells transfected with hY3 compared with cells not transfected (ns). **d** *IL13* mRNA levels were measured in cells stimulated with PMA and ionomycin for 24 h compared to cells not stimulated. mRNA levels were assessed by RT-qPCR (*p < 0.05; **p < 0.01; ***p < 0.001)

Since allergic asthma is associated with the presence of IL-13-producing cells in bronchoalveolar lavage (BAL) [18] and plays a relevant role in the development of the disease, we decided to study the *IL13* gene expression in our model by using qPCR (Fig. 4).

As mentioned above, we analyzed *IL2* mRNA levels as a control to monitor that the Jurkat cells were correctly stimulated. Although both *IL2* and *IL13* could be detected at low levels in the non-stimulated cells (Fig. 4a, c) their levels substantially increase upon stimulation (Fig. 4b, d). Moreover, *IL13* RNA significantly increased in those cells transfected with hY3 compared to the non-transfected (*p* = 0.041) (Fig. 4d).

We also measured the cytokine levels secreted to the culture media by ELISA. After 24 h of stimulation, IL-13 levels were below the detection limit, while IL-2 levels were around 600 pg/mL. The differences observed in the levels of the secreted cytokines can be due to different cues for their production in Jurkat cells, as has been discussed for other cytokines [20]. The low IL-13 production might depend on the intrinsic characteristics of this cell line, although changes in *IL13* mRNA levels have been extensively described [3, 5].

**Fig. 5:**
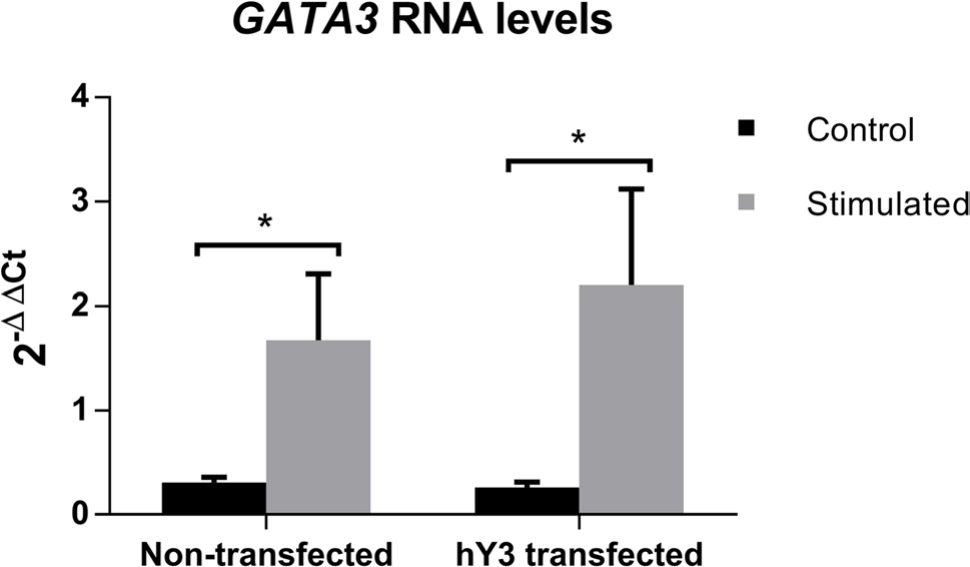
*GATA3* mRNA levels measured by RT-qPCR in cells stimulated with PMA and ionomycin for 24 h compared to cells not stimulated in control cells and cells transfected with hY3 (*p < 0.05)

To determine whether the increase of *IL13* transcripts observed in hY3-transfected cells upon stimulation responded to an increase in transcription, we studied the expression of the transcription factor GATA3 in our experimental conditions. GATA3 is a master regulator of T_H_2 cells that is upregulated during T_H_2 differentiation; it regulates *IL13* expression by binding to its promoter, and their levels substantially increase upon stimulation [25]. Interestingly, we did not detect any significant change in *GATA3* expression when comparing hY3-transfected cells with non-transfected cells upon stimulation (Fig. 5). This result might suggest a role of hY3 in *IL13* mRNA stabilization rather than in *IL13* transcription.

### hY3 is present in Jurkat-derived exosomes

The presence of Y-RNAs in extracellular fluids, such as saliva or blood serum, seems to be associated with ribonucleoprotein complexes or extracellular vesicles, suggesting a role in signal transmission, amplification, or modulation of different responses in their target cells [6]. Considering this, a set of experiments was designed to detect *RNY3* RNA in exosomes from Jurkat cell culture medium.

**Fig. 6:**
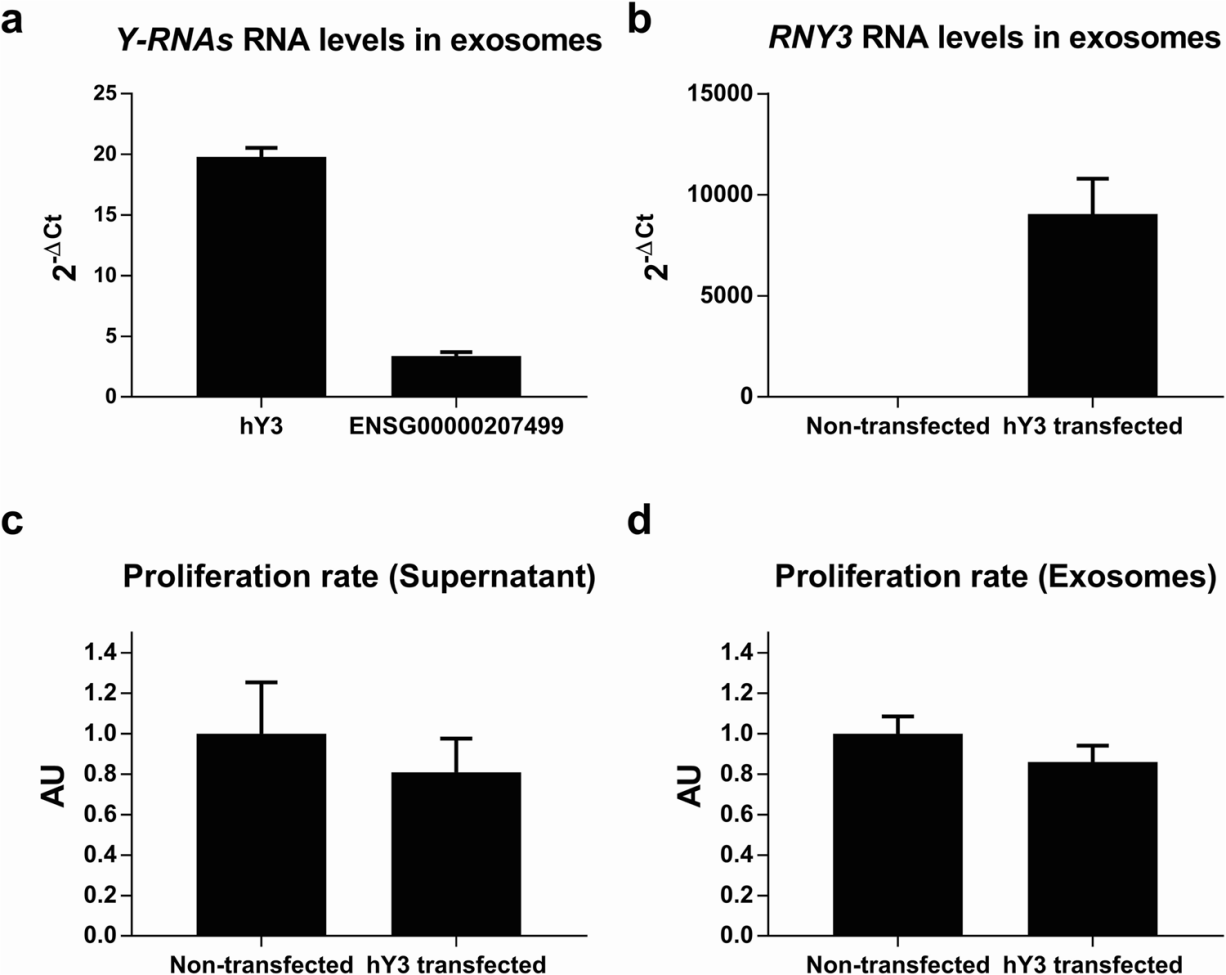
hY3 presence in Jurkat-derived exosomes. **a** Comparison of hY3 and ENSG00000207499 in Jurkat-derived exosomes. **b** hY3 RNA levels in Jurkat-derived exosomes after transfection with hY3. **c** Proliferation index in cells incubated for 24 h with the supernatants of control cells and cells transfected with hY3. **d** Proliferation index in cells incubated for 24 h with exosomes purified from supernatants of control cells and cells transfected with hY3. RNA levels were assessed by RT-qPCR. Proliferation was assessed by BrdU incorporation. (AU, arbitrary units; *p < 0.05)

We purified exosomes from the medium after 72 h of incubation. We detected hY3 at high abundance when normalized with *GAPDH* (Fig. 6a). In fact, the exosome hY3 level was higher than that of other Y-RNAs, such as *ENSG00000207499* (Fig. 6a). Next, we investigated whether hY3 transfection would lead to an increase in exosomes, purifying them from the medium 48 h post-transfection. Transfected cells showed a 12.8-fold increase in hY3 expression compared to the mock control. Interestingly, we also found an exponential enrichment in hY3 in the purified exosomes (Fig. 6b).

As mentioned above, exosomes may be a vehicle to transfer hY3 signaling to target cells. To check this hypothesis, we incubated Jurkat cells for 24 h with conditioned media from both control cells and hY3-transfected cells and also with exosomes isolated from those media, and found a reduction in the proliferation index of the cells incubated with hY3-enriched exosomes and the media containing them (Fig. 6c, d). However, the differences in proliferation did not reach significance (*p* = 0.112). Furthermore, stimulation of Jurkat cells incubated with hY3-enriched exosomes caused no significant effect on proliferation (Supp. Fig. 2).

## Discussion

Allergic diseases, particularly allergic asthma, have increased over the last decades, becoming a public health burden. Interest and need to understand the mechanisms underlying these diseases have become increasingly urgent. As in other multifactorial diseases, the development of allergic diseases not only resides on heredity or genetic predisposition but also on other factors, i.e., environmental conditions and epigenetic modulation, that have become crucial components to understanding the onset and evolution of the disease. In this regard, understanding the role of non-coding RNAs in asthma is currently a hot topic. Previous works by our group found significant overexpression of three Y-RNAs (*ENSG00000207499*, *ENSG00000201228*, and *ENSG00000201555*) in allergic asthmatic patients [11, 23]. Unlike miRNAs, little is known about the function of these small non-coding RNAs in these patients. Their sequence homology to the highly conserved Y-RNA *RNY3* led us to evaluate the effect of increasing *RNY3* RNA levels using the Jurkat cell line as an in vitro model for lymphocyte T stimulation. When cells were stimulated with PMA and ionomycin, hY3 levels increased significantly. Using this stimulation strategy, we could observe a significant increase in *IL13* mRNA levels in cells transfected with synthetic *RNY3* RNA compared to cells with hY3 basal levels. Furthermore, we detected increased cell proliferation upon stimulation in hY3-transfected cells. In addition, we could confirm that Jurkat-derived exosomes contained hY3 RNA molecules, that hY3 transfection produces an increase of hY3 in exosomes, and finally, according to the trend in the diminution of the proliferation observed, that these exosomes seem to have a minor effect over cell proliferation.

The study of T cell signaling in Jurkat cells is well established and has been extensively reported [1]. It has also been used for studying T cell activation in allergy. Here, we used this model to disentangle the role of hY3 upon cell stimulation, finding an increase in hY3 levels upon stimulation with phorbol 12-myristate 13-acetate and ionomycin (Fig. 2). This result aligns with our previous studies, where we observed an overexpression of three Y-RNAs in allergic patients [11, 23].

Numerous studies have shown different functions of Y-RNAs. Several models have shown that Y-RNAs act both in favor of and against cell proliferation since they are involved in DNA replication and cell cycle progression [17]. Although we could not detect any significant differences in cell proliferation when hY3 was overexpressed, the significant increase in cell proliferation upon stimulation (Fig. 3c) might suggest a possible role in the cellular response to stimulation.

Accordingly, *IL2* and *IL13* transcripts significantly increased upon stimulation, as previously described [15]. Interestingly, a significant increase of *IL13* transcripts upon stimulation was found when hY3 levels were increased upon transfection (Fig. 4d). Notwithstanding, no changes were observed in the expression of the *IL13* transcription factor *GATA3*, suggesting that *IL13* transcription is not affected by hY3 (Fig. 5). It is crucial to consider several circumstances to establish how hY3 can modify *IL13* transcript levels. First, hY3 is part of ribonucleoprotein complexes and binds to proteins involved in mRNA processing and splicing, such as HuR (ELAVL1) [16]. Interestingly, HuR is involved in T cell activation response and differentiation [13, 31]. Also, Karginov et al. showed that HuR produced a broad control through upstream factors during T cell activation and that its depletion produced NFAT1 upregulation and NFAT2 downregulation [13]. Both NFAT2 and NFAT1 are critical regulators of early gene transcription in response to T cell activation and can bind to *IL13* promoter in Jurkat cells [22]. Finally, hY3 has been proposed as a molecular sponge for HuD (ELAVL4) activity, an RNA-binding protein that plays a fundamental role during neuronal differentiation controlling neuronal cell fate [12]. hY3 competes with HuD target mRNAs and limits HuD access to the polysomal compartment [27]. Considering this, we could speculate that hY3 could act as a molecular sponge of HuR, impairing its interactions with other transcripts. Moreover, although HuR does not impact *NFAT2* expression through its 3′ UTR [13], an association of *NFAT5* mRNA with HuR has been found [7]; thus, we cannot rule a similar relation for *NFAT1*.

In addition to the role of Y-RNAs inside the cell, extracellular vesicles containing Y-RNAs have been found in different biological fluids and related to several diseases, including different types of cancer and cardiovascular diseases [6, 8]. In fact, Driedonks et al. recently showed that most full-length Y-RNAs are present in extracellular vesicles [5]. These vesicles seem to be a vehicle for mediating intercellular RNA transfer that could alter gene expression in target cells [33].

We have detected hY3 transcripts in exosomes derived from Jurkat cells, confirming what was previously reported elsewhere [14]. Moreover, exosomes derived from cells transfected with hY3 contained high levels of this transcript (Fig. 6b). Despite not being statistically significant, cell proliferation seemed impaired when growing in a medium supplemented with exosomes derived from hY3-overexpressing Jurkat cells (Fig. 6d). Nevertheless, no effect could be detected upon stimulation. This observation suggests that Y-RNAs can be transported in exosomes, but whether these exosomes have a function other than stimulating proliferation remains to be further explored and opens a new avenue in T cell signaling research.

Our study presents some limitations. First, we used the immortalized cell line Jurkat E6-1 as a model of T cell, which might not be as good a proxy to T_H_2 cell response as a primary T cell culture. Additionally, concerning *IL13*, only mRNA and not secreted protein could be detected at confident levels. Nevertheless, we could see increased *IL13* and *GATA3* expression levels upon cell activation, characteristic of the T_H_2 response. Finally, our observations over exosomes and hY3 were made to study the mere effect of Y-RNA content on T cell proliferation.

In conclusion, we have shown the effect of hY3 modulation in our T cell model and observed its influence on the cell proliferation and *IL13* mRNA levels upon stimulation. Because IL-13 is a pivotal cytokine in T2-type asthma, this study opens up new ways to study the potential regulatory function of hY3 over IL-13 production and its implications for asthma development.

## Supplementary Information

Below is the link to the electronic supplementary material.

Supplementary file1 (JPG 542 KB) **Supplementary Figure 1. **Protein extracts of purified exosomes. Protein extracts of purified exosomes were subjected to WB to assess exosome purification. Lanes 1-3 Jurkat-derived exosomes of non-transfected cells, lanes 4-6 Jurkat-derived exosomes of hY3 transfected cells. Western blots were hybridized with polyclonal antibodies against CD9, CD81 and CD63 as specified by the manufacturer of the kit.

Supplementary file2 (JPG 631 KB) **Supplementary Figure 2. **Cell stimulation in the presence of purified exosomes. (a) *RNY3* RNA levels after transfection, (b) *RNY3* RNA levels in Jurkat-derived exosomes after transfection, (c) Comparison of proliferation rates of cells in the presence of *RNY3* enriched exosomes and control exosomes upon stimulation and in normal conditions. No significative differences were found associated with hY3 content exosomes (p = 0.317 and 0.252 in control and stimulation conditions, respectively). RNA levels were assessed by RT-qPCR. Proliferation was assessed by BrdU incorporation. (AU, arbitrary units; *, p < 0.05; **, p < 0.01; ***, p < 0.001)

## Declarations

### Disclaimer

The funders had no role in the design of the study, in the collection, analyses, or interpretation of data, in the writing of the manuscript, or in the decision to publish the results.
