# Supplementary figures and images for: RNY3 modulates cell proliferation and IL13 mRNA levels in a T lymphocyte model: a possible new epigenetic mechanism of IL-13 regulation
Source: J Physiol Biochem. 2022 Sep 12;79(1):59–69. doi: 10.1007/s13105-022-00920-6 (PMC9905197; doi:10.1007/s13105-022-00920-6)

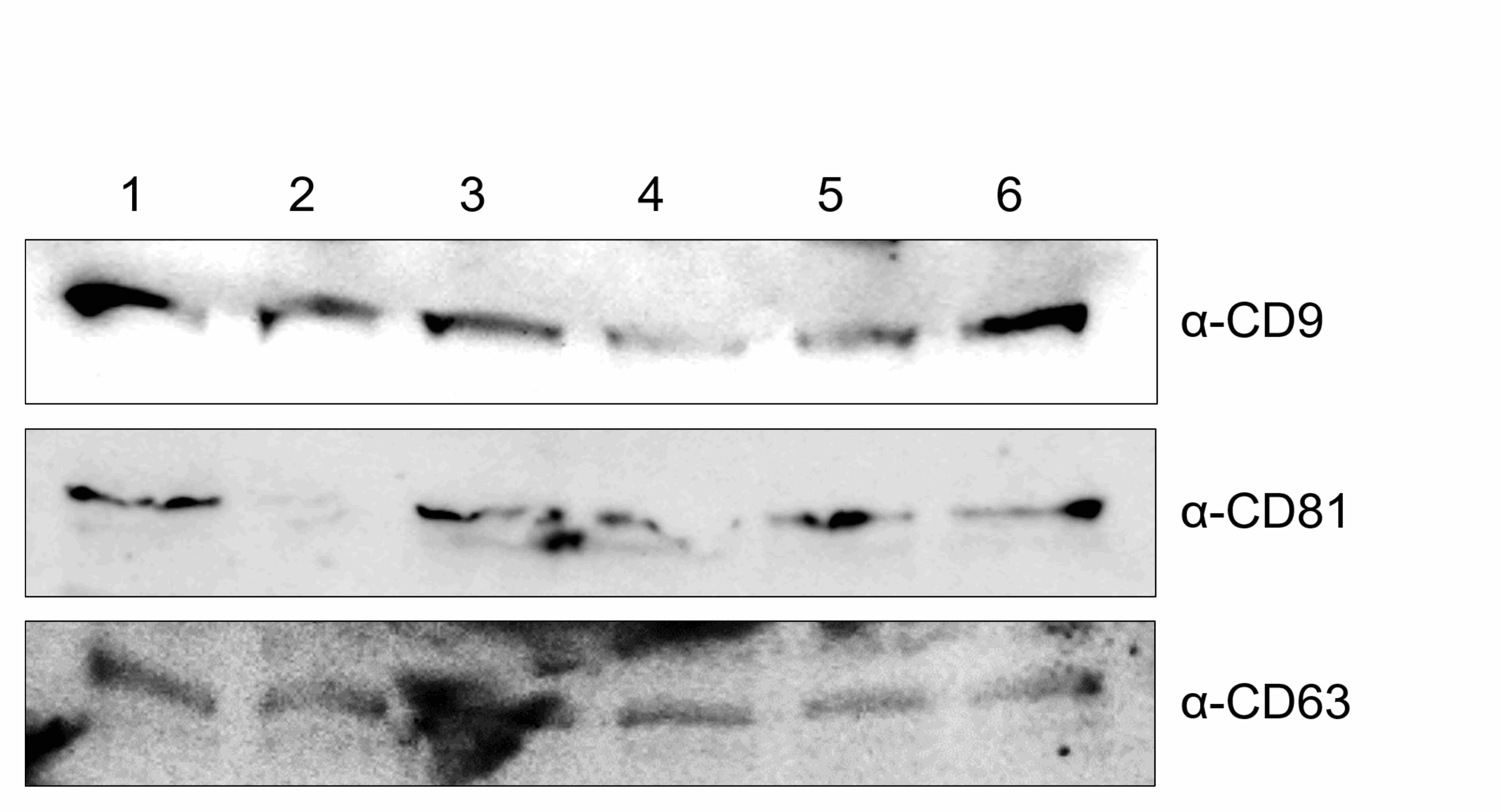

Supplement: Supplementary file 1 — Supplementary file1 (JPG 542 KB) Supplementary Figure 1. Protein extracts of purified exosomes. Protein extracts of purified exosomes were subjected to WB to assess exosome purification. Lanes 1-3 Jurkat-derived exosomes of non-transfected cells, lanes 4-6 Jurkat-derived exosomes of hY3 transfected cells. Western blots were hybridized with polyclonal antibodies against CD9, CD81 and CD63 as specified by the manufacturer of the kit. [file 13105_2022_920_MOESM1_ESM.jpg]

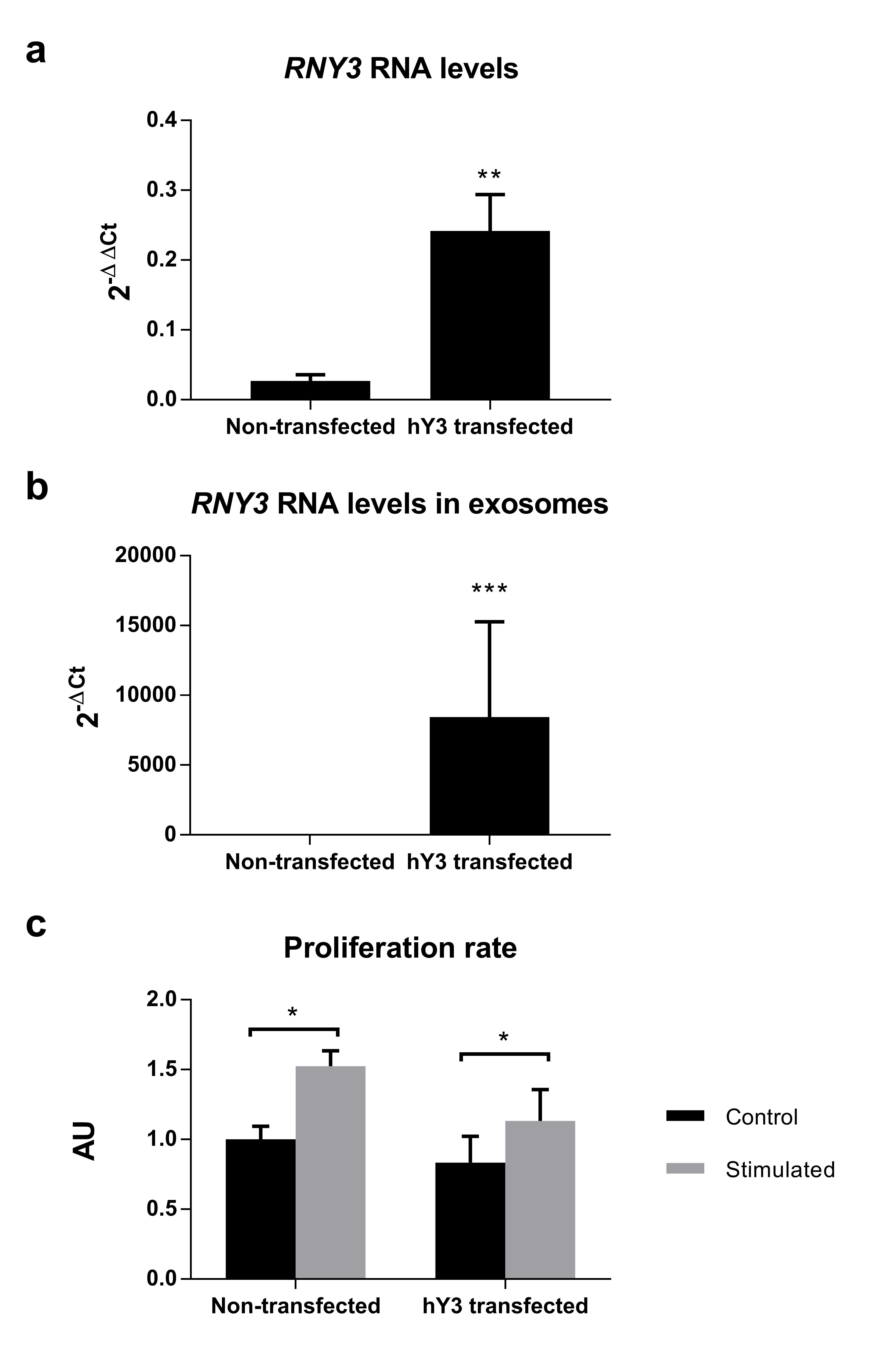

Supplement: Supplementary file 2 — Supplementary file2 (JPG 631 KB) Supplementary Figure 2. Cell stimulation in the presence of purified exosomes. (a) RNY3 RNA levels after transfection, (b) RNY3 RNA levels in Jurkat-derived exosomes after transfection, (c) Comparison of proliferation rates of cells in the presence of RNY3 enriched exosomes and control exosomes upon stimulation and in normal conditions. No significative differences were found associated with hY3 content exosomes (p = 0.317 and 0.252 in control and stimulation conditions, respectively). RNA levels were assessed by RT-qPCR. Proliferation was assessed by BrdU incorporation. (AU, arbitrary units; *, p < 0.05; **, p < 0.01; ***, p < 0.001) [file 13105_2022_920_MOESM2_ESM.jpg]
